# Manganese–Iron-Supported Biomass-Derived Carbon Catalyst for Efficient Hydrazine Oxidation

**DOI:** 10.3390/molecules31020354

**Published:** 2026-01-19

**Authors:** Karina Vjūnova, Huma Amber, Dijana Šimkūnaitė, Zenius Mockus, Aleksandrs Volperts, Ance Plavniece, Galina Dobele, Aivars Zhurinsh, Loreta Tamašauskaitė-Tamašiūnaitė, Eugenijus Norkus

**Affiliations:** 1 Center for Physical Sciences and Technology (FTMC), Sauletekio Avenue 3, LT-10257 Vilnius, Lithuania; karina.vjunova@ftmc.lt (K.V.); huma.amber@ftmc.lt (H.A.); dijana.simkunaite@ftmc.lt (D.Š.); zenius.mockus@ftmc.lt (Z.M.); 2 Latvian State Institute of Wood Chemistry, Dzerbenes Str. 27, LV-1006 Riga, Latvia; aleksandrs.volperts@kki.lv (A.V.); galina.dobele@kki.lv (G.D.); aivarsz@edi.lv (A.Z.)

**Keywords:** manganese, iron, nitrogen-doped carbon, hydrothermal carbonization, hydrothermal synthesis, hydrazine oxidation

## Abstract

This study presents a straightforward strategy for producing novel, effective and inexpensive functional non-noble metal-supported carbon materials made from abundant natural biomass. These materials offer a cost-effective alternative to noble metals for the oxidation of hydrazine (HzOR) and demonstrate the potential for widespread adoption of green, energy-saving hydrazine-based technologies in energy applications. Highly efficient and cost-effective iron (Fe) and manganese–iron (MnFe)-supported nitrogen-doped carbon (N–C) materials were developed using hydrothermal synthesis. Meanwhile, the N–C material was obtained from biomass—birch-wood chips—using hydrothermal carbonisation (HTC), followed by activation and nitrogen doping of the resulting hydrochar. The morphology, structure, and composition of the MnFe, MnFe/N–C, and Fe/N–C catalysts were determined using scanning electron microscopy (SEM), X-ray diffraction (XRD), and energy dispersive X-ray spectroscopy (EDS). The activity of the catalysts for HzOR in an alkaline medium was evaluated using cyclic voltammetry (CV). Depositing MnFe particles onto N–C was shown to significantly enhance electrocatalytic activity for HzOR compared to the Fe/N–C catalyst and especially to the MnFe particles catalyst in terms of highly developed porous structure, which offers the largest surface area, lowest onset potential, and highest current density response, resulting in the strongest catalytic activity. These results suggest that the MnFe/N–C catalyst could be a highly promising anode material for HzOR in direct hydrazine fuel cells (DHFCs).

## 1. Introduction

In recent years, there has been significant scientific focus on finding viable alternatives to fossil fuels. In the face of fossil fuel scarcity and environmental pollution, fuel cells are the most promising technological development for facilitating the advanced use of clean fuels and renewable energy sources [1,2]. Direct hydrazine fuel cells (DHFCs) are an innovative and green energy technology with the potential to transform future energy demand. Thanks to Daihatsu’s pioneering efforts in using such systems to power vehicles, including small cars and trucks, they have already demonstrated impressive results [3]. This technology offers several distinct advantages. DHFCs use a carbon-free fuel with a relatively high hydrogen content and produce a carbon-free end product (only N_2_ and H_2_O). They also have zero carbon emissions, a high theoretical cell voltage of 1.56 V, high power and energy density, a wide operating pH range, and a low thermodynamic potential of −0.33 V for the hydrazine oxidation reaction (HzOR) [4,5]. This latter characteristic of HzOR makes it an ideal alternative to replace the sluggish oxygen evolution reaction (OER) in green hydrogen production via water splitting, which requires a thermodynamic potential of 1.23 V [6]. Replacing OER with HzOR, which has a lower oxidation potential, is a promising approach to energy-saving and cost-efficient hydrogen production [7,8,9,10,11,12]. Despite the many advantages of DHFCs, they still face significant performance challenges, particularly with regard to overvoltage and kinetics. The lack of efficient, cost-effective HzOR catalysts is a significant barrier to the widespread use of DHFCs. The operation of DHFCs is based on HzOR at the anode (Equation (1)) and oxygen reduction reaction (ORR) at the cathode (Equation (2)):

Anode: N_2_H_4_ + 4OH^−^ → N_2_ + 4H_2_O + 4e^−^     E_0_ = −1.21 V (1)

Cathode: O_2_ + 2H_2_O + 4e^−^ → 4OH^−^     E_0_ = 0.40 V (2)

Equation (3) describes the overall process occurring in DHFCs:

N_2_H_4_ + O_2_ → N_2_ + 2H_2_O     E_0_ = 1.61 V (3)

N_2_H_4_ can be electrochemically oxidized as a neutral molecule in basic solutions (Equation (1)), with the final products of its electro-oxidation being environmentally friendly and harmless N_2_ and H_2_O. The efficiency of various noble metals, including platinum [13,14], palladium [15], gold [16], and silver [17], as well as their alloys (e.g., AuPd [18]), as catalysts for the HzOR has been well documented in the literature. However, importantly, DHFCs do not require the use of prohibitively expensive noble metals as electrocatalysts. This is a distinct advantage over hydrogen–air and direct alcohol fuel cells. Recently, the combination of noble metals and 3d transition metals (such as Mn, Fe, Ni, Co, and Cu) has been extensively researched in electrocatalysis. For example, NiO_x_-Pt [19], Co-Ru [20], Pd-MnFe_2_O_4_ [21], Co-Pt_3_ [22], Cu-Pt [23], manganese–vanadium oxide, and Au nanoparticle-modified graphene oxide nanosheets [24] or carbon nanotubes [25] have been developed and investigated. However, the high cost and scarcity of these noble metals make them unsuitable for the large-scale production of electrocatalysts. In contrast, earth-rich transition metals incorporated into precious metals are low-cost and improve the electrocatalytic process due to their high conductivity, numerous active sites, multiple oxidation states, and superior activity. Additionally, the alkaline HzOR is one of the reactions for which non-precious metal catalysts are highly competitive, even outperforming precious metals [26]. This explains why many recent studies have focused on 3d transition metals. These studies have led to the development of highly efficient, durable, and cost-effective binary or ternary catalysts composed of non-precious metals, including Mn-doped Ni-Co [27], Fe/P-NiMoO_4_ [28], NiCoFe_3_O_4_ [29], NiFe_2_O_4_ [30], and Fe-Ni alloy [31]. Improvements in the performance of HzOR can be attributed to the modification of the electronic properties of the catalysts. However, the detailed mechanism and the principles for screening the alloying elements are still uncertain.

Of these elements, Fe and Mn are of particular interest due to their abundance in natural resources, benign environmental impact, non-toxicity, wide range of oxidation states, and potential to regulate charge distribution and facilitate electron transfer at the surface. These catalysts are often used in combination with carbon materials or heteroatom-doped carbons (N, P, O, S), such as MnO/C [32], Fe/N–C [33], and Mn-doped Ni-Co HNS/CNT [27]. Carbon-based catalysts are of great importance due to their high conductivity, chemical stability, high specific surface area, controlled porosity, and large number of electroactive sites. The incorporation of metal nanoparticles on a carbon support has been demonstrated to enhance catalytic activity by restructuring the metal phase and facilitating electron transfer [34,35]. Additionally, doping carbon structures with elements such as N, P, O, or S makes these materials highly electrocatalytically active. The incorporation of heteroatom dopants facilitates a redistribution of charge around the adjacent carbon atoms, changing the electronic configuration of the catalyst surface and creating a large number of active catalytic sites in a multilayer porous structure [34,36]. It has been highlighted that the control and optimization of the types and densities of heteroatom dopants (especially N-atoms in the form of pyridinic and graphitic N species) and the design of material structures (surface area and porosity) are essential steps to obtaining heteroatom-doped carbon materials with good electrocatalytic efficiency for the HzOR [37]. These materials have attracted considerable attention due to their numerous distinctive advantages, including greater stability and improved catalytic performance towards the electrooxidation of hydrazine [37,38,39].

However, despite the advantages offered by N, P, O, or S carbon structures, the performance of catalysts for HzOR is still primarily limited by relatively sluggish reaction kinetics due to strongly bound intermediates, competition from undesirable side reactions, limited availability of active sites, and stability issues within the catalysts themselves. In addition, most of these high-performance conventional carbon materials are derived from fossil fuels using energy-intensive, lengthy synthetic processes that are not only environmentally unfriendly, but also quite expensive, making their commercialization non-viable. In this context, biomass-derived carbon has emerged as a highly promising alternative to conventionally produced carbon materials for the next generation of energy storage and conversion systems [40,41]. Biomass is an attractive carbon precursor in terms of its renewability, earth-abundance, environmental friendliness, low cost, non-toxicity, sustainability, ease of production, and the variety of heteroatoms (N, P, S, etc.) in its intrinsic composition [40,42]. In this respect, wood and wood derivatives have great potential as a resource for advanced materials for energy storage and clean energy production [43,44].

Recently, ionic liquid-modified cellulose nanowhiskers (CNWs) were used as precursors to prepare heteroatom (N and S)-doped nanostructured carbon catalysts for HzOR [45]. The resulting material showed efficient electrocatalytic activity for the hydrazine oxidation reaction, with an onset potential close to the thermodynamic value of the reaction. This value was found to be superior to those obtained for other related materials. The outstanding catalytic activity of the material was attributed to the heteroatom dopants and defect sites in the materials formed during carbonisation by the liquid placed around the CNWs. Furthermore, the specific nature of the dopant-associated chemical moieties and vacancy sites created in the material played a significant role in enhancing the electrocatalytic activity of the material towards the reaction. In addition, the authors of [46] demonstrated that the use of cellulose filter paper, functioning as both precursor and self-template, enabled the synthesis of nanoporous carbon catalysts that exhibited efficient electrocatalytic activity towards the HOR with low overpotential and high current density. Furthermore, the double heteroatom doping strategy involving carbon derived from bone char and N- and S-heteroatoms has been shown to be a successful method to achieve a high surface area, optimal density of heteroatom dopant groups, and defect sites containing a metal-free electrocatalyst with electrocatalytic activity. This makes them the most efficient electrocatalysts for HzOR that have ever been reported for this reaction [47]. These metal-free biomass-derived catalysts have the potential to replace the conventional metal-based catalysts that are commonly used in HOR and related reactions. A significant focus is currently being directed towards heteroatom-doped biomass-derived electrocatalysts that additionally incorporate earth-abundant transition metals and feature advanced characteristics. These are primarily used as electrocatalysts in a variety of applications, including water splitting, re-chargeable batteries, fuel cells, and supercapacitors [48]. However, the application of metal-doped biomass-derived carbon catalysts for HzOR remains under-researched, with only a few studies having been conducted thus far [49,50,51]. To the best of our knowledge, no research has yet been carried out on Mn coupled with Fe within multicomponent systems on N-doped carbon, particularly derived from biomass, for HzOR specifically. It is imperative to emphasize that Fe-based materials are particularly appealing for nanostructured HzOR electrocatalysts. For example, a N-doped carbon matrix supporting iron–molybdenum carbide (Fe_2_MoC) nanoparticles yielded the earliest onset potential for any carbon-supported HzOR catalyst at the time (0.28 V vs. RHE; see Figure 4c in Ref. [52]). Moreover, the Fe–Mn nanostructures in the form of manganese ferrite (MnFe_2_O_4_) exhibit unique magnetic properties, high chemical stability, and excellent biocompatibility [21]. These characteristics render them highly versatile for a wide range of applications, extending beyond the field of catalysis; most notably, they find application in biomedicine and environmental remediation, as well as in electronic and magnetic devices [21]. In view of these findings, investigating the potential of Fe–Mn on N-doped carbon derived from a biomass system as a HzOR electrocatalyst is reasonable.

In addition, a considerable number of high-performance biomass derived carbon catalysts are produced using complex, time-consuming methods (e.g., pyrolysis, hydrothermal carbonization and physicochemical activation), which are often difficult to scale up for industrial use [53]. Therefore, there is an ongoing need for simpler, cost-effective, and feasible synthesis strategies. This study presents a simple strategy to preparing a novel, effective and inexpensive functional carbon-based catalyst from abundant natural biomass, offering a cost-effective alternative to noble metals for HzOR. The findings demonstrate the innovation and potential of biomass-derived catalysts in developing green catalytic techniques, as well as their relevance for energy applications. In this study, we used the N-doped carbon (denoted as N–C) as a substrate for depositing MnFe or Fe particles using a hydrothermal synthesis method. The N–C was prepared by the hydrothermal carbonization of birch wood, followed by activation with NaOH and N-doping as described in Reference [54]. The activity of resulting material for HzOR was then studied.

## 2. Results

### 2.1. Characterization of Catalysts Morphology and Structure

**Figure 1:**
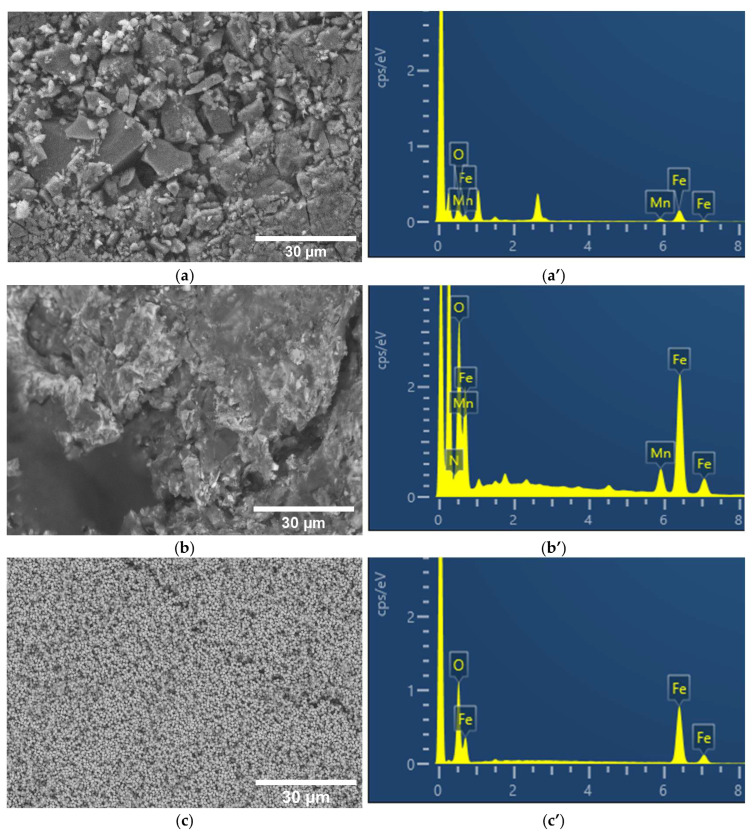
The SEM images of the MnFe (**a**), MnFe/N–C (**b**), and Fe/N–C (**c**) catalysts, which were obtained through hydrothermal synthesis. The corresponding EDS spectra of MnFe (**a’**), MnFe/N–C (**b’**), and Fe/N–C (**c’**).

The synthesis of MnFe and MnFe or Fe particles supported on N–C composites was accomplished via hydrothermal synthesis. The N–C substrate was successfully prepared from biomass by subjecting birch chips to hydrothermal carbonization, followed by activation with NaOH at 800 °C and nitrogen doping described in Ref. [54]. It was determined that the N–C material used had a large specific surface area of 2431 m^2^ g^−1^ and a micro–mesoporous structure with more than 50% of the mesopore volume [54]. Due to the doping, the nitrogen content in the carbon material was 4.60%, of which 57.61% was in the pyridinic form [54]. Figure 1 shows SEM images for pure MnFe particles (a), MnFe particles supported on N–C (b), and Fe particles supported on N–C (c).

The corresponding X-ray energy-dispersive spectroscopy (EDS) spectra of the MnFe, MnFe/N–C, and Fe/N–C catalysts are given in Figure 1a’–c’. The MnFe particles are irregularly shaped and consist of a mixture of large angular flakes and small granular particles (Figure 1a). While some particles have sharp edges and flat surfaces, suggesting crystalline structures, others appear more amorphous or agglomerated. The larger flakes range in size from 2 to 10 µm, while the smaller particles or agglomerates are likely to be in the sub-micron to ~1 µm range.

This indicates a broad distribution of particle sizes. In the case of MnFe particles supported on N–C (Figure 1b), the surface appears rough, porous, and heterogeneous, indicating the presence of a carbon matrix embedded with metal particles. The MnFe particles appear well-dispersed. The MnFe particles (bright spots) are embedded in structures that are typical of doped carbon materials, such as flaky or wrinkled surfaces (Figure 1b). In the case of Fe particles supported on N–C (Figure 1c), Fe nanoparticles are homogeneously dispersed on the N–C support. The composition of the MnFe, MnFe/N–C, and Fe/N–C catalysts was confirmed using EDS and is given in Table 1. The corresponding EDX spectra show that the prepared catalysts contain Mn or/and Fe and O. Strong Fe-Kα (~6.4 keV) and Fe-K_β_ peaks, as well as Mn-Kα (~5.9 keV) and large O-K peaks (~0.52 keV), are evidence in the EDS spectra and indicate that the synthesized materials are in oxidized form, likely Fe-Mn oxide or Fe oxide.

The results revealed that the MnFe/N–C catalyst contained 4.92 at% Mn, 29.50 at% Fe, and 65.58 at% O, while the MnFe catalyst contained 6.74 at%, 33.60 at%, and 59.65 at% Mn, Fe, and O, respectively. The Fe/N–C catalyst contained 47.80 at% Fe and 52.20 at% O. The Mn:Fe:O molar ratios were found to be 1:5:8.9, 1:6:13.3, and 0:1:1.1 for MnFe, MnFe/N–C, and Fe/N–C, catalysts, respectively.

**Figure 2:**
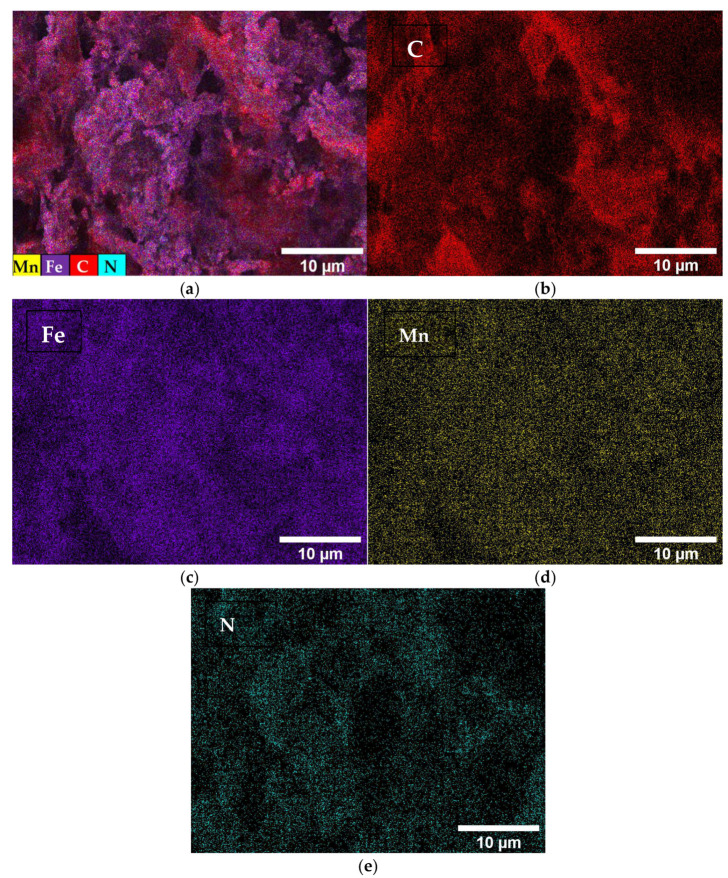
(**a**) The survey map of the MnFe/N–C catalyst. Maps (**b**–**e**) show the distribution of the individual elements: C (**b**), Fe (**c**), Mn (**d**), and N (**e**).

The distribution of the Mn, Fe, N, and C elements in the MnFe/N–C catalyst was also analyzed. Figure 2a shows an overview map of the elemental distribution in the MnFe/N–C catalyst.

Figure 2b–e show maps of each element: C, Fe, Mn, and N. As can be seen, the elements Fe, Mn, C, and N are evenly distributed across the entire surface of the catalyst.

**Figure 3:**
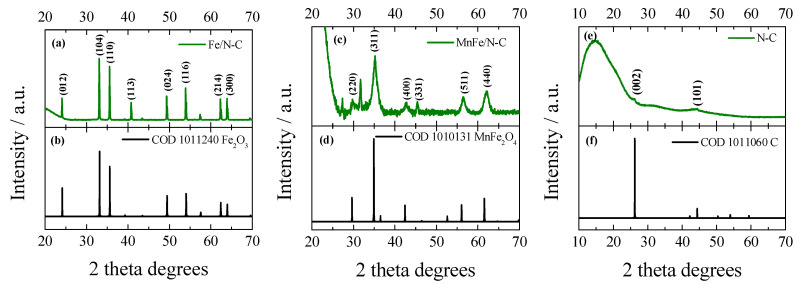
XRD patterns of the Fe/N–C (**a**), MnFe/N–C (**c**), and N–C (**e**) catalysts. Peak positions are indicated according to Crystallography Open Database (COD) data cards: Fe_2_O_3_—1011240 (**b**); MnFe_2_O_4_—1010131 (**d**); C—1011060 (**f**).

Figure 3 shows the XRD patterns of Fe/N–C (a), MnFe/N–C (c), and N–C (e) catalysts. The XRD pattern of the Fe/N–C catalyst shows diffraction peaks at 2θ values of 24.13°, 33.12°, 35.61°, 40.83°, 49.42°, 54°, 62.39°, and 63.96°, which correspond to the lattice plane of (012), (104), (110), (113), (024), (116), (214), and (300), respectively, of rhombohedral structure of iron(III) oxide (Fe_2_O_3_) hematite (COD card no. 1011240) (Figure 3a). For the MnFe/N–C catalyst, the formation of the cubic spinel structure of the manganese ferrite (MnFe_2_O_4_) is confirmed by peaks at 2θ values 29.91°, 35.19°, 42.65°, 45.43°, 56.4°, and 62.04°, which correspond to the lattice planes (220), (311), (400), (331), (511), and (440), respectively, as shown in Figure 3c and in accordance with the COD card no. 1010131. Furthermore, the (311) plane is indicative of the cubic spinel structure and is essential for confirming the formation of MnFe_2_O_4_ [55].

Figure 3e shows the XRD pattern of N–C, which exhibits a broad diffraction peak at around 2θ ≈ 26°, corresponding to the (002) plane of turbostratic carbon, and a weak peak at ~44° assigned to the (101) plane (COD 1011060) (see Figure 3e,f). These broad peaks indicate a predominantly amorphous carbon structure with low graphitization, attributed to nitrogen doping and abundant structural defects. Furthermore, the interlayer spacing (d002) calculated using Bragg’s law (see Section 3.2, “Characterization of Catalysts”) was approximately 0.356 nm, which is larger than that of pristine graphite (0.335 nm). This expansion of the interlayer distance is attributed to lattice distortion and structural defects introduced by nitrogen doping. The coexistence of the carbon (002) peak with MnFe_2_O_4_ reflections implies a hybrid structure, in which crystalline MnFe_2_O_4_ is supported by or partially embedded within the N-doped carbon matrix. XRD analysis confirmed the formation of the MnFe_2_O_4_ spinel phase (COD 1010131), SEM images revealed the catalyst morphology, and EDS spectra qualitatively showed the presence of Fe, Mn, and O, which is consistent with the spinel composition.

### 2.2. Determination of the Electrochemically Active Surface Areas (ECSAs)

**Figure 4:**
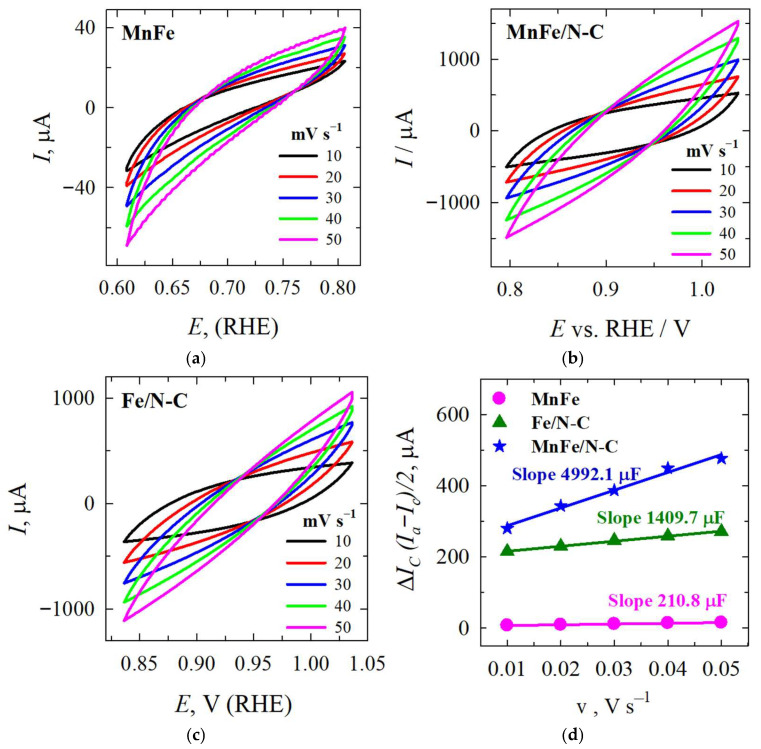
CVs of the MnFe (**a**), MnFe/N–C (**b**), and Fe/N–C (**c**) catalysts in an N_2_-saturated 1 M KOH in the non-faradaic potential region at various scan rates (10–50 mV/s) (**d**).

In order to determine the ECSAs of the catalysts, the capacitance method was used. Cyclic voltammograms (CVs) were recorded for tested materials at various scan rates within the electrode potential range in the non-faradaic potential region. This method relies on measuring the double-layer capacitance (C_dl_). Figure 4 shows CVs of the MnFe (a), MnFe/N–C (b), and Fe/N–C (c) catalysts in a N_2_-saturated 1 M KOH solution at various scan rates (5, 10, 20, 30, 40, 50 mV s^−1^). Calculated C_dl_ and ECSA values of the synthesized MnFe, Fe/N–C, and MnFe/N–C catalysts were presented in Table 2.

Significantly greater C_dl_ and ECSA values were observed for the Fe and MnFe particles supported on N-doped carbon than that for the use unsupported MnFe catalyst (Table 2). The C_dl_ values were found to be 210.8, 1409.7, and 4992.1 μF for the MnFe, Fe/N–C, and MnFe/N–C catalysts, respectively (Figure 4d). The calculated ECSA values were found to be 5.3, 35.2, and 124.8 cm^2^ for the MnFe, Fe/N–C, and MnFe/N–C, respectively. The calculated ECSA values for the Fe and MnFe particle catalysts supported on N–C were 6.6 and 23.5 times higher, respectively, than the ECSA value for the unsupported MnFe catalyst. The determined roughness factor (R_f_) of the catalysts shows that the ECSA of the MnFe, MnFe/N–C, and Fe/N–C catalysts is 2.6, 17.6, and 62.4 times greater than the geometric area, respectively. The large ECSA of the MnFe/N–C and Fe/N–C catalysts allows the creation of a greater number of active sites, which contributes to faster charge/mass transport processes and higher HzOR electrocatalytic activity.

### 2.3. The Oxidation of Hydrazine

**Figure 5:**
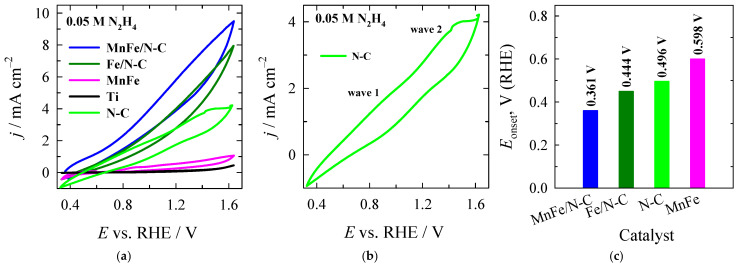

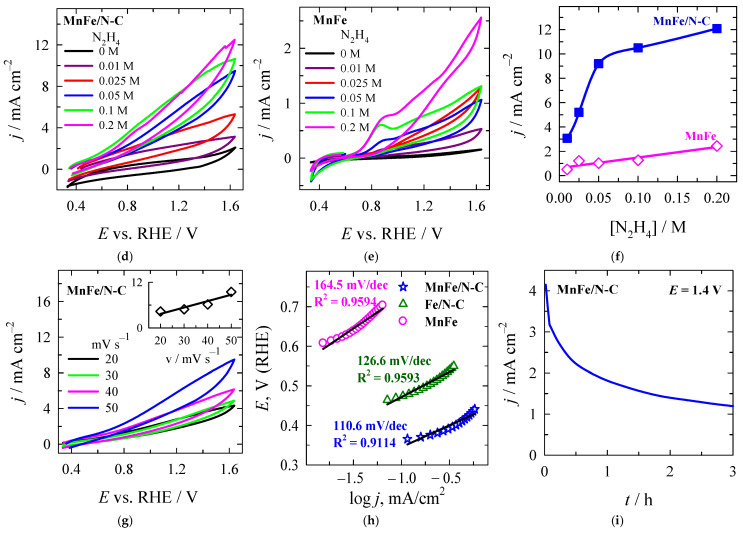
Comparison of CVs (**a**) and onset potentials (**c**) recorded on the MnFe/N–C, MnFe, Fe/N–C, N–C, and bare Ti catalysts in a 0.05 M N_2_H_4_ + 1 M KOH solution at a scan rate of 50 mV s^−1^. (**b**) CV recorded on N–C in the 0.05 M N_2_H_4_ + 1 M KOH solution. CVs recorded on the MnFe/N–C (**d**) and MnFe (**e**) catalysts in a 1 M KOH solution containing 0.01–0.2 M N_2_H_4_ concentrations at 50 mV s^−1^. (**f**) Dependence of the N_2_H_4_ oxidation current densities at MnFe/N–C and MnFe at a potential of +1.6 V on N_2_H_4_ concentration. (**g**) CVs recorded on the MnFe/N–C catalyst at different scan rates (20–50 mV s^−1^) in the 0.05 M N_2_H_4_ + 1 M KOH solution. The inset represents the dependence of current density at +1.6 V on scan rate. (**h**) Tafel slopes obtained from the data in (**a**). (**i**) Chronoamperometric curve of the MnFe/N–C catalyst at a constant potential of +1.4 V in a 0.05 M N_2_H_4_ + 1 M KOH solution.

The oxidation of hydrazine was studied using cyclic voltammetry (CV). Figure 5a shows the CVs for pure N–C, titanium (Ti), MnFe, Fe/N–C, and MnFe/N–C catalysts, recorded in a solution of 0.05 M N_2_H_4_ + 1 M KOH at 25 °C with a scan rate of 50 mV s^−1^. As shown in Figure 5a, the current densities observed on the pure Ti electrode are negligible in the hydrazine solution. It is evident that these values are considerably lower than those observed on the N–C support. In addition, the current value response on the latter indicates some difference in the shape of the CV curve, pointing to the development of two hardly discernable waves (Figure 5b). There are two transfer processes involved in the electrochemical oxidation of hydrazine. One of these is the adsorption of hydrazine oxidation products, and the other is the formation of a passive, non-conductive layer of intermediate products obtained from the oxidation process on the electrode surface [56,57,58]. It is supposed that the saturation of active sites may occur due to the presence of N_2_H_4_ molecules and their oxidation intermediates that reduce the number of places available for later adsorption. Thus, the overall rate of hydrazine oxidation decreases and the current density remains the same. It is therefore suggested that two distinct active sites are present, and/or that hydrazine undergoes stepwise/partial oxidation at different potentials [58]. Comparing the CVs recorded on the MnFe/N–C, MnFe, Fe/N–C, N–C, and bare Ti catalysts in a 0.05 M N_2_H_4_ + 1 M KOH solution at a scan rate of 50 mV s^−1^ shows that MnFe or Fe particles supported on the nitrogen-doped carbon and even the N–C support itself exhibit significantly higher electrocatalytic activity for the oxidation of N_2_H_4_ than an unsupported MnFe particle catalyst (Figure 5a). The low catalytic current response of the latter catalyst suggests reduced active sites and sluggish electron transfer kinetics, which are apparently due to the aggregated nature of the catalyst. Moreover, the oxidation of N_2_H_4_ starts at more negative electrode potentials at the MnFe/N–C catalyst at ca. +0.361 V vs. RHE compared to the Fe/N–C (+0.444 V), N–C (+0.496 V), and MnFe (+0.598 V) catalysts (Figure 5c), indicating a high electrocatalytic activity of this catalyst towards HzOR due to the synergistic effect between the properties of MnFe and the N–C support.

The measured oxidation current density values of N_2_H_4_ at an electrode potential of +1.6 V are approximately 4, 7, and 9 times higher on the N–C, Fe/N–C, and MnFe/N–C catalysts, respectively, than on the MnFe catalyst (see Figure 5a). As mentioned above, this high activity of the MnFe/N–C and Fe/N–C catalysts can be attributed to the synergistic effect of Mn, Fe, and N–C, as well as the highly developed porous structure of N–C, which has a specific surface area of 2431 m^2^ g^−1^ and the advisable proportion of mesopores volume to total volume of 57%, facilitating mass transfer and, therefore, diminishing the influence of diffusion issues [59]. Furthermore, N–C contains 4.60% of the nitrogen, most of which is in the pyridinic form (57.61%), thus creating active sites for hydrazine adsorption.

Figure 5d,e show the CVs that were recorded on the MnFe/N–C and MnFe catalysts, respectively, in a 1 M KOH solution and that contain 0.01–0.2 M N_2_H_4_ at a scan rate of 50 mV s^−1^. Notably, there is no clear sign of an oxidation current within the potential window under study in the 1 M KOH background solution. Notwithstanding, the CV curves show no clearly defined peaks within the studied potential region, while the XRD data indicate the presence of Mn^2+^ and Fe^3+^ ions in the catalysts in the form of MnFe_2_O_4_ and Fe_2_O_3_. Consequently, the transformation of these ions in alkaline media by the following reactions cannot be entirely ruled out [55]:

MnFe_2_O_4_ + 2OH^−^ ↔ MnOOH + 2FeOOH + 2e^−^ (4)

FeOOH + OH^−^ ↔ FeO_2_^−^ + H_2_O + e^−^ (5)

Fe + 2OH^−^ ↔ Fe(OH)_2_ + 2e^−^ (6)

3Fe(OH)_2_ + 2OH^−^ ↔ Fe_3_O_4_ + 4H_2_O + 2e^−^ (7)

The current density values for N_2_H_4_ oxidation on both catalysts increase with increasing N_2_H_4_ concentration (see Figure 5d,e). The absence of a cathodic peak, coupled with the presence of an anodic peak, confirms the irreversible nature of the charge transfer process. An almost linear increase in current density was observed at the MnFe catalyst across the entire examined N_2_H_4_ concentration range, as well as at the MnFe/N–C catalyst for lower concentrations ranging from 0.01 to 0.5 M (see Figure 5f). This indicates the presence of active surface sites on both modified electrodes. However, the role of intermediates in affecting the availability of redox sites capable of interacting with a greater number of molecules should also be taken into account. As demonstrated in Figure 5f, there is less significant increase in the anodic peak current at concentrations above 0.5 M for the MnFe/N–C catalyst. This result is consistent with the data referenced in Ref. [56], in which a dual dependence of the current density on the hydrazine concentration was identified. Consequently, 0.5 M N_2_H_4_ can be regarded as the optimum concentration. Beyond this concentration, the adsorption of oxidation products at the surface of the MnFe/N–C electrode may impede the further oxidation of hydrazine molecules to a potentially greater extent. Furthermore, a discernible deviation from the linear response has been observed at elevated N_2_H_4_ concentrations, which is likely attributable to kinetic limitation, as previously suggested in Ref. [56]. Additionally, the impact of the blocking effect of active sites by the electroactive substance or intermediates is substantiated by the CVs’ dependence on successive cycles for each concentration presented in Figure S1 (Supplementary Material). It is also important to note that two distinct active sites develop more clearly on the pure MnFe catalyst, especially at higher hydrazine concentrations. This behaviour may be attributed to the adsorption of intermediates on the remaining active sites, thereby hindering further oxidation of N_2_H_4_ [56].

Meanwhile, significantly higher current densities were recorded for N_2_H_4_ oxidation on the MnFe/N–C catalyst than on the MnFe catalyst (Figure 5f). These increased more sharply at lower N_2_H_4_ concentrations ranging from 0.01 to 0.05 M on the MnFe/N–C catalyst, suggesting that numerous active surface sites are available for hydrazine oxidation. This results in the production of a large amount of N_2_ gas through the oxidation reaction of N_2_H_4_ (Equation (1)) in this concentration region. Subsequently, the mass transfer process of N_2_H_4_ possibly will be improved due to the stirring action caused by the evolution of N_2_ gas. However, a slightly lower increase in current densities was observed on this catalyst at higher N_2_H_4_ concentrations, starting from 0.05 M, possibly due to a change in the N_2_H_4_ concentration near the electrode surface, due to the adsorption of intermediates [56]. In contrast, there was negligible increase in current densities on pure MnFe. On average, the current density values determined for N_2_H_4_ oxidation on the MnFe/N–C catalyst were approximately 5 times higher than those on the unsupported MnFe catalyst across all concentration regions. This indicates that the MnFe/N–C catalyst has an abundance of active sites on its surface. This enables a greater amount of N_2_H_4_ to be oxidized to nitrogen via these sites, resulting in a higher current density observed on CVs.

Figure 5g shows CVs recorded on the MnFe/N–C catalyst in a solution of 0.05 M N_2_H_4_ and 1 M KOH at different scan rates (20–50 mV s^−1^). As shown in Figure 5g, the current density values on the MnFe/N–C catalyst increase as the scan rate raises from 20 to 50 mV s^−1^, indicating faster charge transfer and higher reaction rates at higher scan rates. The inset plot in Figure 5g shows a nearly linear relationship between current density and scan rate. This is characteristic of a surface-controlled process. N_2_H_4_ oxidation on the MnFe/N–C catalyst predominantly occurs at or near the electrode surface and is likely governed by surface adsorption rather than diffusion of N_2_H_4_ from the bulk electrolyte. Higher scan rates accelerate electron transfer and suppress the accumulation of adsorbed intermediates. This maintains high surface coverage of N_2_H_4_ on the Mn–Fe active sites. These processes promote rapid N–H bond cleavage and enhance the kinetics of hydrazine oxidation, confirming strong adsorption of N_2_H_4_ on the MnFe/N–C catalyst.

The Tafel slopes calculated were within the range of approximately 110.6 to 164.5 mV dec^−1^. The MnFe/N–C catalyst had the lowest Tafel slope (110.6 mV dec^−1^) compared to the Fe/N–C catalyst (126.6 mV dec^−1^) and the MnFe catalyst (164.5 mV dec^−1^) (see Figure 5h). These surfaces catalyze the HzOR with different Tafel slopes, suggesting different HzOR rates on different catalysts. Meanwhile, the lowest Tafel slope determined for the MnFe/N–C catalyst means that it can achieve much faster charge transfer kinetics across the catalytic interface at lower potentials and higher current densities than other catalysts during hydrazine oxidation. Furthermore, the Tafel slopes for the N–C-supported Fe and MnFe catalysts were found to be in good agreement with the reported slope values for a single-electron transfer rate-determining step [60], indicating that electrooxidation proceeds through a single-electron rate-determining step. HzOR involves the stepwise dehydrogenation of intermediates that are adsorbed onto the catalyst surface. The key steps are as follows [30,61,62]:

N_2_H_4_ + OH^−^ → N_2_H_3_* + H_2_O + e^−^ (8)

N_2_H_3_* + OH^−^ → N_2_H_2_* + H_2_O + e^−^ (9)

N_2_H_2_* + OH^−^ → N_2_H* + H_2_O + e^−^ (10)

N_2_H* + OH^−^ → N_2_ + H_2_O + e^−^ (11)

Computational studies suggest that the first step of the dehydrogenation of N_2_H_4_ (Equation (4)) is rate-determining for carbon-supported single-atom catalysts (SACs), such as Fe, Mn, Co, Ni, and Cu [63,64,65]. Moreover, DFT calculations indicated that the dehydrogenation process of N_2_H_4_ was more thermodynamically favourable on the Fe–N sites of Fe–NC than that on the pyridinic-N sites of NC. This enhances the HzOR activity of Fe–NC by improving the HzOR-related intermediate dehydrogenation process [65]. Based on these studies and the determined Tafel slopes, it is supposed that the N_2_H_4_ dehydrogenation step by Equation (8) is rate-determining for N–C-supported Fe and MnFe catalysts. This suggests a faster dehydrogenation process with additional pyridinic-N sites in the catalyst composition compared to the unsupported MnFe catalyst.

Additionally, the electrooxidation reaction process can be coupled with non-faradaic hydrazine decomposition reactions, as shown in the following Equations (12) and (13):

N_2_H_4_ → N_2_ + 2H_2_ (12)

3N_2_H_4_ → N_2_ + 4NH_3_ (13)

Coupling Fe-based catalysts with other transition metals has been shown to catalyze HzOR due to the promotion of OH^−^ ion adsorption, which is believed to initiate the slow N_2_H_4_ electrooxidation step according to Equation (8). At the same time, the fast steps are accelerated according to Equations (9)–(11) [66]. Another possible explanation is that these catalysts can inhibit the non-faradaic decomposition of hydrazine [31]. Furthermore, electroactive metal centres, such as Fe(III) and Mn(II), can act as redox centres in the oxidation of hydrazine. Additionally, the ability of Fe(II)/Fe(III) and Mn(II)/Mn(III) to undergo redox cycling could enhance hydrazine oxidation [60]. However, these faradaic or non-faradaic side reactions may have a different impact on the contribution of each component of the catalyst material, revealing different reasons for the catalyst activity and potentially having a detrimental effect on HzOR.

To provide more detailed information about the MnFe/N–C catalyst, a long-term durability test was performed in a solution of 0.05 M N_2_H_4_ + 1 M KOH at an applied potential of +1.4 V for 3 h, as illustrated in Figure 5i. The good stability of the catalysts can be assigned to the synergistic effect of Mn and Fe ions in the N–C catalyst structure. In addition, it results from the effective role of N–C in the nanocatalyst structure, which increases the active surface area and conductivity also. However, by-products of hydrazine oxidation possibly could accumulate on the surface of the MnFe/N–C catalyst, reducing the adsorption of hydrazine molecules on the active sites and thus reducing its efficiency slightly.

Table 3 presents a comparison of the HzOR activity of some recently reported electrocatalysts alongside values determined in this study.

The obtained onset potential (E_onset_) values for HzOR of +0.361 V is higher compared to Ni-Fe/NF (−0.110 V) [31], Fe_2_MoC@NC (+0.280 V) [52], Fe_3_C@NCNTs (+0.270 V) [67], MnO/N–C (+0.286 V) [32], NiCoFe_3_O_4_ (+0.315 V) [29], SeNCM-1000 (+0.340 V) [68], and Fe–NC-2–1000 (+0.350 V) [65], but lower compared to the E_onset_ values for porous carbon-derived from filter paper (+0.378 V) [46], N–C (+0.397 V) [32], NiFe_3_O_4_ (+ 0.595 V) [29], Fe-MOF (+0.604 V) [29], Fe_2_O_3_/ECP-15 (+0.610 V) [69], and CoFe_3_O_4_ (+0.657 V) [29]. This clearly shows that the synthesized MnFe and Fe catalysts using N-doped carbon derived from biomass are comparable to those listed in Table 3.

The successful synthesis of high-performance MnFe and Fe catalysts can be attributed to a combination of material properties and optimized synthesis methods. This primarily results in strong synergistic bimetallic interactions between the two metals, thereby enhancing chemical reactivity. Additionally, the interaction between Mn and Fe oxides may also potentially promote the formation of lattice defects and oxygen vacancies, which could act as highly active sites accelerating key reaction steps of HzOR. Furthermore, optimized synthesis methods and the use of specific nitrogen-doped carbon supports with thermal and chemical stability result in the development of materials with high specific surface areas and pore volumes. This leads to better dispersion of active sites and maximized contact between reactants and the catalyst surface, thus enhancing catalytic activity.

## 3. Materials and Methods

### 3.1. Synthesis of Metal Particles Supported Nitrogen-Doped Carbon (M/N–C)

**Figure 6:**
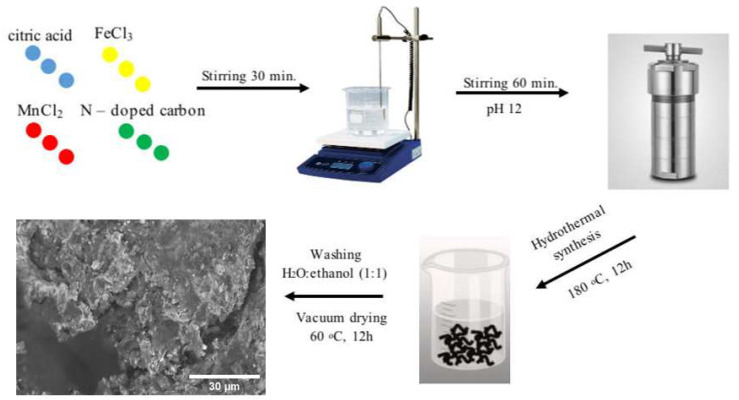
Scheme for the synthesis of MnFe/N–C catalyst.

Nitrogen-doped carbon derived from biomass—birch wood was used as a substrate for depositing MnFe and Fe nanoparticles. The synthesis of N–C was described in detail in the Ref. [54]. The pure MnFe, MnFe/N–C, and Fe/N–C catalysts were obtained by hydrothermal synthesis. Briefly, the synthesis of MnFe/N–C involved a reaction mixture containing 0.6487 g of FeCl_3_ (Chempur, Piekary Śląskie, Poland), 0.3958 g of MnCl_2_·4H_2_O (Chempur, Piekary Śląskie, Poland), 1.43 g of citric acid (Alfa Aesar, Ward Hill, MA, USA), and 100 mg of N–C in a total volume of 30 mL. The mixture was stirred on a magnetic stirrer for 30 min. The pH of the solution was then adjusted to pH 12 using a 10 M NaOH (Chempur, Piekary Śląskie, Poland) solution. After this, the mixture was stirred for a further 60 min. The resulting homogeneous solution was transferred to a 50 mL Teflon liner, placed in a stainless steel autoclave, and heated at 180 °C for 12 h. The synthesized black particles (MnFe/N–C) were then washed several times with a C_2_H_5_OH:H_2_O (1:1, *v*/*v*) (Chempur, Piekary Śląskie, Poland) solution and dried under vacuum at 60 °C for 12 h. Figure 6 shows the synthesis scheme of the MnFe/N–C catalyst.

The MnFe catalyst was synthesized using the same reaction mixture, but without the addition of N–C. The Fe/N–C catalyst was synthesized using the same reaction mixture, but without the addition of MnCl_2_·4H_2_O.

### 3.2. Characterization of Catalysts

The surface morphology and elemental composition of the catalysts were evaluated using a Hitachi Ltd. (Tokyo, Japan) TM 4000 Plus scanning electron microscope. The powder samples were mounted on conductive copper tape on aluminum stubs. SEM imaging was performed in high-vacuum mode at an accelerating voltage of 15 kV, a working distance of 10.4 mm and a magnification of 3000×. The BSE (backscattered electrons) detector was used for the MnFe/N–C and Fe/N–C samples, and the SE detector was used for the MnFe sample. Elemental analysis was carried out using an Oxford Instruments AZtecOne EDS system (High Wycombe, UK) at 15 kV with a live time of 60 s. A total of three SEM images from three different areas were analyzed. The reported composition of the catalysts represents their average composition.

The XRD patterns of the catalyst powders under study were measured using a D2 PHASER X-ray diffractometer (Bruker, Karlsruhe, Germany), which was equipped with a LYNXEYE XE-T detector and Cu Kα radiation (λ = 1.54060 Å). The diffractometer operated at 30 kV and 10 mA. Measurements were conducted in step scan mode, with a step size of 0.041° (on the 2θ scale) and a counting time of 2 s per step within the 2θ range of 5–90°. Phase identification was performed using Crystallography Open Database (COD) data cards. The interlayer spacing (d002) for the N–C material was calculated using Bragg’s law according to Equation (14):

nλ = 2d sin θ,(14)

where n is the order of the reflection, λ is the wavelength of the incident X-ray, d is the distance between crystal planes, and θ is the angle of incidence at which the X-rays strike the crystal planes.

### 3.3. Investigation of Hydrazine Oxidation

The efficiency of hydrazine oxidation was evaluated using cyclic voltammetry (CV) in an alkaline medium (1 M KOH (Chempur, Piekary Śląskie, Poland) solution). Measurements were carried out using a potentiostat/galvanostat PGSTAT100 (Metrhom, Herisau, Switzerland) and a standard three-electrode electrolytic cell, where the MnFe/N–C, MnFe, Fe/N–C catalysts coated on a titanium (Ti) electrode with a geometric surface area (GSA) of 2 cm^2^ was used as the working electrode. A Pt sheet and an Ag/AgCl (3 M KCl) electrode were used as the counter and reference electrodes, respectively. Cyclic voltammograms (CVs) were recorded in 1 M KOH solution containing 0.025–0.2 M hydrazine (Chempur, Piekary Śląskie, Poland) at a scan rate of 50 mV s^−1^. The electrode potential was evaluated against the reversible hydrogen electrode (RHE). Current densities were normalized to the GSA of the catalysts. Inks containing the following catalysts were prepared by ultrasonically mixing 10 mg of each catalyst in a C_2_H_5_OH:H_2_O (1:1, *v*/*v*) solution, with a total volume of 2 mL, and 20 µL of 5 wt.% Nafion (Ion Power Inc., Tyrone, PA, USA): MnFe/N–C, MnFe, Fe/N–C, and N–C. Then, 200 µL of the prepared ink was pipetted onto the Ti surface. The catalyst loading was 500 µg cm^−2^.

The stability of the MnFe/N–C catalyst was investigated using chronoamperometry (CA) by recording the chronoamperometric curve on the catalyst at a constant potential value of +1.4 V over a period of three hours.

The electrochemically active surface areas (ECSAs) of the catalysts were determined by measuring the double-layer capacitance (C_dl_). CVs were recorded on the investigated catalysts at various scan rates in the non-faradaic region, and the slope of the resulting curve was calculated by plotting the difference in the anodic and cathodic currents against the scan rate [70,71,72]. The charging current, I_c_, of the electrodes at each scan rate was determined from the CVs via the following Equation (15):

I_c_ [A] = (I_anodic_ − I_cathodic_)OCP (15)

The C_dl_ values were evaluated by plotting a graph of the charging current against the scan rate and calculating the slope, as shown in Equation (16):

Slope = C_dl_ [F] = ΔI_C_ [A]/Δν [V s^−1^] (16)

Then, the ECSA values were calculated using a specific capacitance (C_s_) of 40 μF cm^−2^ [70,71,72] and an Equation (17):

ECSA [cm^2^] = C_dl_ [μF]/C_s_ [μF cm^−2^] (17)

The roughness factor (R_f_) was determined according to the following Equation (18):

R_f_ = ECSA/GSA (18)

where GSA is the geometrical surface area of the catalysts.

## 4. Conclusions

Fe- and MnFe-supported nitrogen-doped carbon catalysts synthesized using simple birch wood as the raw material via hydrothermal carbonization were successfully used as efficient, noble metal-free, nanoporous, carbon-based electrocatalysts for the oxidation of hydrazine in an alkaline medium. The presence of abundant exposed active sites due to MnFe doping, the large specific surface area of the nitrogen-doped carbon for facilitated mass transfer, and the synergistic effect between Mn, Fe, and N–C in the prepared MnFe/N–C catalyst result in outstanding activity for hydrazine oxidation compared to the other catalysts under study. It significantly outperforms the Fe/N–C catalyst and especially the MnFe catalyst in terms of its highly developed porous structure, which offers the largest surface area, lowest onset potential, and highest current density response, resulting in the strongest catalytic activity. These results suggest that the MnFe/N–C catalyst could be a highly promising anode material for HzOR in DHFCs. Moreover, the synthesis strategy for non-noble metal catalysts proposed in this study offers a new, simple, and environmentally friendly approach to producing inexpensive, highly efficient anode catalysts for developing efficient direct hydrazine fuel cells.

## Figures and Tables

**Table 1:** Surface composition of the catalysts determined by EDS analysis.

Sample	Element, at%			Molar Ratio, at%
	Mn	Fe	O	Mn:Fe:O
MnFe	6.74	33.60	59.65	1:5:8.9
MnFe/N–C	4.92	29.50	65.58	1:6:13.3
Fe/N–C	–	47.80	52.20	0:1:1.1

**Table 2:** Capacities (C_dl_) and ECSA values of the synthesized catalysts.

Sample	C_dl_, µF	ECSA, cm^2^	R_f_
MnFe	210.8	5.3	2.6
Fe/N–C	1409.7	35.2	17.6
MnFe/N–C	4992.1	124.8	62.4

**Table 3:** Comparison of HzOR activity of some recently reported electrocatalysts.

Sample	Electrolyte	E_onset_, V vs. RHE	Ref.
MnO/N–C	1 M KOH + 0.1 M N_2_H_4_	+0.286	[32]
N–C	1 M KOH + 0.1 M N_2_H_4_	+0.397	[32]
Ni-Fe/NF	1 M KOH + 0.5 M N_2_H_4_	−0.110	[31]
Fe–NC-2–1000	1 M KOH + 0.1 M N_2_H_4_	+0.350	[65]
Fe_2_MoC@NC	1 M KOH + 0.1 M N_2_H_4_	+0.280	[52]
NiCoFe_3_O_4_	1.0 M KOH + 0.5 M N_2_H_4_	+0.315	[29]
NiFe_3_O_4_	1.0 M KOH + 0.5 M N_2_H_4_	+0.595	[29]
CoFe_3_O_4_	1.0 M KOH + 0.5 M N_2_H_4_	+0.657	[29]
Fe-MOF	1.0 M KOH + 0.5 M N_2_H_4_	+0.604	[29]
Fe_3_C@NCNTs	1.0 M KOH + 0.1 M N_2_H_4_	+0.270	[67]
SeNCM-1000	1.0 M KOH + 0.1 M N_2_H_4_	+0.340	[68]
Porous carbon-derived from filter paper (PCDFs-900)	0.1 M PBS (pH 7.4) + 0.05 M N_2_H_4_	+0.378	[46]
Fe_2_O_3_/ECP-15	1.0 M KOH + 0.1 M N_2_H_4_	+0.610	[69]
Fe/N–C	1.0 M KOH + 0.05 M N_2_H_4_	+0.444	This study
MnFe/N–C	1.0 M KOH + 0.05 M N_2_H_4_	+0.361	This study

## Data Availability

The original contributions presented in this study are included in the article/Supplementary Material. Further inquiries can be directed to the corresponding author(s).

## Supplementary Materials

The following supporting information can be downloaded at https://www.mdpi.com/article/10.3390/molecules31020354/s1, Figure S1: Continuous CVs of hydrazine oxidation recorded at MnFe/N–C catalyst in a 1 M KOH solution containing N_2_H_4_ concentration of (a) 0.01 M, (b) 0.025 M, (c) 0.05 M, (d) 0.1 M, and (e) 0.2 M. Scan rate 50 mV s^−1^.
