# Supplementary material for: Manganese–Iron-Supported Biomass-Derived Carbon Catalyst for Efficient Hydrazine Oxidation
Source: Molecules. 2026 Jan 19;31(2):354. doi: 10.3390/molecules31020354 (PMC12844456; doi:10.3390/molecules31020354)
Supplement: Supplementary file 1 [file molecules-31-00354-s001.zip › molecules-4039432-supplementary.pdf]

Supplementary Materials

Manganese–iron-supported biomass-derived carbon catalyst for efficient hydrazine oxidation

Karina Vjūnova [1], Huma Amber [1], Dijana Šimkūnaitė [1], Zenius Mockus [1], Aleksandrs Volperts [2], Ance Plavniece [2], Galina Dobele [2], Aivars Zhurinsh [2], Loreta Tamašauskaitė-Tamašiūnaitė [1,*], and Eugenijus Norkus [1,*]

[1] Center for Physical Sciences and Technology (FTMC), Sauletekio ave. 3, LT-10257 Vilnius, Lithuania; karina.vjunova@ftmc.lt (K.V.); huma.amber@ftmc.lt (H.A.); dijana.simkunaite@ftmc.lt (D.S.); zenius.mockus@ftmc.lt (Z.M.)

[2] Latvian State Institute of Wood Chemistry, Dzerbenes Str. 27, LV-1006 Riga, aleksandrs.volperts@kki.lv; galina.dobele@kki.lv; aivarsz@edi.lv

* Correspondence: loreta.tamasauskaite@ftmc.lt (L.T.-T.); eugenijus.norkus@ftmc.lt (E.N.)

Results and Discussion

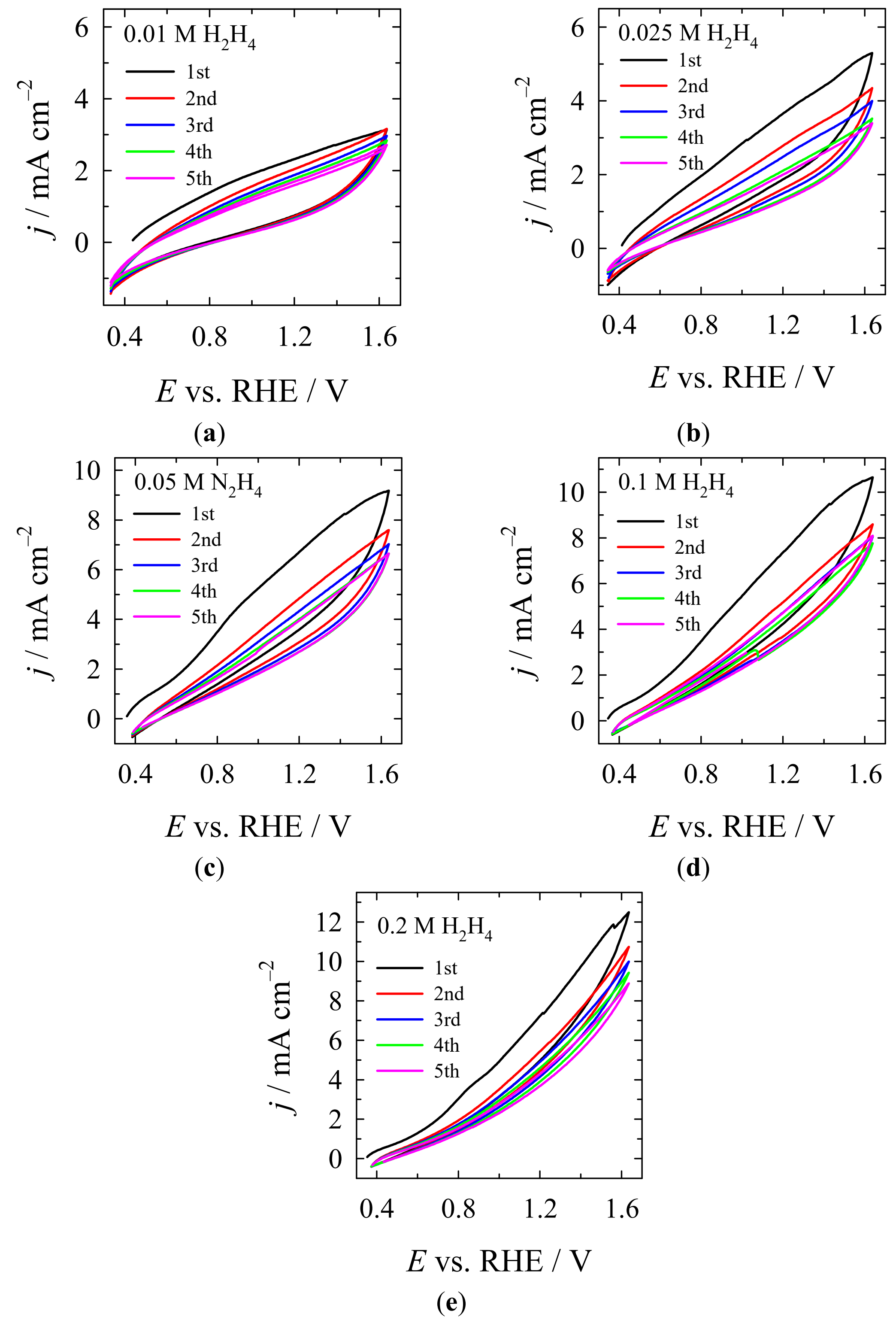

Figure S1. Continuous CVs of hydrazine oxidation recorded at MnFe/N-C catalyst in a 1 M KOH solution containing N_2H_4 concentration of: (**a**) 0.01 M, (**b**) 0.025 M, (**c**) 0.05 M, (**d**) 0.1 M, and (**e**) 0.2 M. Scan rate 50 mV s^{-1}.
